# Assessing the role of attentional inhibition and cognitive flexibility in convergent and divergent thinking during creative task performance

**DOI:** 10.1186/s40359-026-04582-7

**Published:** 2026-04-29

**Authors:** Zoe D. Hughes, Linden J. Ball, Jeannie Judge

**Affiliations:** 1 School of Psychology, Liverpool John Moores University, Liverpool, United Kingdom; 2 School of Engineering and Computing, University of Lancashire, Preston, United Kingdom; 3 School of Psychology and Humanities, University of Lancashire, Preston, United Kingdom

**Keywords:** Creative cognition, Attentional inhibition, Cognitive flexibility, Convergent thinking, Divergent thinking

## Abstract

Findings regarding the role of attentional inhibition and cognitive flexibility in creative cognition have been inconsistent, potentially due to methodological differences in measures of attention and creativity. To clarify these relationships, we conducted two experiments assessing how attentional inhibition and cognitive flexibility relate to convergent and divergent creative thinking. In Experiment 1, individual differences in flanker task performance (reaction time and accuracy) across congruent, incongruent and reversed trials were used to derive metrics of inhibition and flexibility, which were then used to predict performance on the Compound Remote Associates Test (CRAT) and Alternative Uses Task (AUT). In Experiment 2, we extended this approach by incorporating the Wisconsin Card Sorting Test to measure cognitive flexibility, and by eliciting subjective reports of problem-solving strategy (i.e., insight vs. analysis) for each CRAT item. Across both experiments, convergent creativity, indexed by CRAT accuracy, was predicted by both attentional inhibition and cognitive flexibility, especially when these supported the use of analytic problem-solving strategies, as measured by retrospective self-report. In contrast, divergent creativity showed weaker and more selective associations, whereby attentional inhibition predicted AUT originality, but not AUT fluency or AUT flexibility, while cognitive flexibility was not significantly related to any AUT dimension. These findings suggest that attentional inhibition and cognitive flexibility demonstrate a stronger and more consistent predictive relationship with convergent thinking performance compared to divergent thinking performance.

## Introduction

Creativity is broadly defined as the capacity to produce ideas that are both novel and appropriate within a specific context [1], with this dual emphasis distinguishing truly creative ideas from responses that are unusual but irrelevant [2, 3]. As a fundamental cognitive capacity underpinning problem-solving, innovation and adaptive behaviour, creativity is widely acknowledged to be a multifaceted construct that arises from a dynamic interplay between neurocognitive systems, thereby occupying a central role across various scientific, artistic, educational, and everyday domains [4–6]. Among these neurocognitive systems, attention has emerged as being core to creative thought, enabling individuals to manage the competing demands of idea generation and idea evaluation by regulating the focus, flexibility, and selectivity of thought [7, 8].

Two attentional capacities that seem particularly relevant to creative problem solving are: (i) attentional inhibition, which functions to suppress irrelevant or incorrect responses as well as to enable access to original associations [9–11]; and (ii) cognitive flexibility, which functions to shift between mental sets and representational frames, allowing for restructuring of problems and the exploration of alternative ideas to reach a solution [12–14]. Although often classified under the broader umbrella of executive functioning, these specific attentional processes may play dissociable roles across different types of creative thinking. However, empirical evidence linking attentional inhibition and cognitive flexibility to creativity remains mixed. Although some studies show positive associations between these attentional processes and creative performance [12, 15, 16], others find no relationships or even negative associations [11, 17–19]. This complex pattern appears to depend on the characteristics of the creativity task employed, such as its degree of structure, its inherent constraints and the extent to which systematic solution evaluation is involved. The current study sought to address these gaps in understanding by examining how individual differences in attentional inhibition and cognitive flexibility predict creative performance across tasks that measure either convergent or divergent thinking, which we describe below.

## Creative cognition and attentional processing

Several theoretical frameworks have been proposed to capture the heterogeneity of the processes that reflect creative cognition. A widely adopted distinction in the creativity literature focuses on the complementary roles of convergent thinking and divergent thinking [20, 21]. Convergent thinking involves the identification of a single correct solution to a problem. It is measured using tasks such as the Remote Associates Test (RAT) and the Compound Remote Associates Test (CRAT), which require participants to find a unifying concept among seemingly unrelated cues (e.g., “age,” “mile,” “sand” → “*stone*” → “stone age”, “milestone”, “sandstone”) [22]. Convergent thinking has been proposed to depend on focused search and the suppression of irrelevant information [23, 24]. In contrast, divergent thinking entails the generation of multiple, varied, and original responses to open-ended problems and is typically assessed using tasks such as the Alternate Uses Task (AUT), in which individuals produce novel uses for common objects (e.g., a brick). Responses are evaluated along multiple dimensions [25, 26], such as fluency (number of ideas), originality (uniqueness of responses) and flexibility (range of responses). Neurocognitive research suggests that divergent thinking is typically associated with increased activity in the default mode network (DMN), linked to spontaneous cognition and associative processing [27, 28], whereas convergent thinking often engages the executive control network (ECN), responsible for goal-directed behaviour, attention, and working memory [12]. Overall, there seems to be an emerging consensus that convergent and divergent thinking tend differentially to engage distinct but interacting systems [8, 16].

Beyond this operational distinction, other frameworks offer additional nuance in understanding the diversity of creative thought. For example, dual-process theories of creativity [24, 29], distinguish between “Type 1” processes (i.e., processes that are intuitive and associative) and “Type 2” processes (i.e., processes that are analytic and deliberate), reflecting similar distinctions that are captured in models of general reasoning [30]. Similarly, a distinction between spontaneous versus deliberate creativity has also been proposed [2], which reflects the degree of executive control involved in creative idea production. Although these frameworks vary in emphasis, they converge on the idea that different creative tasks impose distinct cognitive demands, with some promoting expansive associative exploration, and with others requiring controlled, analytical problem-solving.

Among the various distinctions in the creativity literature, the convergent–divergent dichotomy remains particularly useful for investigating the cognitive mechanisms underpinning creative performance [14, 16]. This is because it offers a task-anchored, process-focused framework that allows researchers to map specific cognitive functions (e.g., attentional inhibition and cognitive flexibility) onto measurable aspects of creative performance. Convergent tasks (e.g., remote associates) appear to be more reliant upon controlled processes, such as sustained attention, attentional inhibition and cognitive flexibility, whereas divergent tasks (e.g., the AUT) reliably index generative fluency and associative breadth [31]. As such, the convergent–divergent typology is utilized in the current series of experiments to provide a theoretically grounded framework for isolating the attentional mechanisms supporting these modes of thinking in creative task performance.

There is still debate about the extent to which creative thinking depends on general executive functions like attentional inhibition and cognitive flexibility, as different theories offer competing views. According to the “business-as-usual model” (see [31] for discussion), creativity emerges from the same top-down, goal-directed processes that support general problem solving and reasoning [32]. Within this framework, executive functions and attentional control are thought to facilitate creative cognition by maintaining task goals, suppressing irrelevant information, and refining ideas during both convergent (solution finding) and divergent (idea generation) tasks [13, 33]. Supporting this view, stronger executive control has been associated with better performance on convergent creativity tasks, including CRAT items, which require constraint satisfaction and solution monitoring [16, 23].

In contrast, the “special-process model” [31] argues that certain forms of creativity are supported by reduced cognitive control or even deliberate disinhibition, particularly divergent thinking and insight problem solving, with the latter reflecting the sudden realisation of a solution that has not been reached through step-by-step analysis [2]. This perspective suggests that a more diffused attentional focus can facilitate access to remote or unconventional associations, which may enhance idea fluency and the likelihood of sudden, intuitive breakthroughs or “Aha!” moments [8, 34]. Indeed, lower inhibitory control has sometimes been linked to greater divergent originality, albeit potentially at the expense of the goal maintenance and interference suppression that are required for successful performance on convergent thinking tasks [11].

A dual-process account arguably offers a more integrative explanation of creativity than the business-as-usual versus special-process dichotomy, by proposing that all creative cognition involves an interaction between associative and executive systems [24, 35]. Within this framework, convergent thinking is thought to rely more continuously on executive processes such as attentional inhibition and cognitive flexibility, which guide structured problem-solving and enable constraint satisfaction [16, 23]. On the other hand, divergent thinking is primarily driven by spontaneous, associative processes, albeit with an element of executive control being recruited to evaluate and select among the generated ideas, sometimes referred to as an “evaluation stage” [7]. Neuroimaging studies lend support to this dual-process account by demonstrating coactivation and functional coupling between the DMN and the ECN during creative tasks, indicative of interactivity between these two systems [27]. Taken together, creative cognition seems to be dependent upon the ability to coordinate associative, generative and evaluative processes that are controlled through attentional mechanisms that enable flexible shifting between problem representations and solution ideas, while also allowing for the suppression of irrelevant information [16, 34].

Given the centrality of attention to creative cognition, recent research has increasingly focused on how specific components of attentional control contribute to distinct aspects of creativity. Attentional inhibition and cognitive flexibility have been identified as key predictors of creative performance that, while sometimes interrelated, play distinct roles in creative cognition, with inhibition enabling suppression of prepotent or irrelevant information, and with flexibility facilitating attentional shifts between ideas, perspectives, or strategies [10, 16, 19]. Emerging evidence suggests that these mechanisms may contribute to creative cognition in differential or even opposing ways depending on the type of creative task being attempted. For example, inhibition may support convergent thinking by enabling focused attention and goal maintenance [36], while flexibility may facilitate divergent thinking by promoting the ability to switch between generation stages (exploratory search and representational change) and evaluation stages (evaluate and select among the generated ideas; cf. [37]).

This latter view is supported by recent meta-analytic findings on mindfulness and creativity, which show that different attentional states that are enhanced through mindfulness practice, such as sustained attention and attentional inhibition, are differentially associated with convergent and divergent thinking [18]. Notably, the meta-analysis found stronger and more consistent benefits of mindfulness for convergent as opposed to divergent thinking. This finding appears to reflect the advantage of sustained attention in supporting evaluative refinement toward a single correct solution in convergent tasks (cf. [38]), compared with divergent tasks, which rely more heavily on idea generation and place less emphasis on the evaluation of ideas. The following sections review in more detail the empirical evidence linking attentional inhibition and cognitive flexibility to creative performance across convergent and divergent tasks.

## Attentional inhibition in creative thinking

To reiterate, attentional inhibition is the ability to suppress irrelevant or automatic stimuli and responses, allowing focus on goal-relevant information [39]. It is considered essential for convergent thinking, which requires narrowing possible solutions to a single correct answer by filtering out misleading associations. The executive attentional framework [40] suggests that inhibitory control supports goal maintenance by preventing interference from task-irrelevant information. This is especially important in tasks like the CRAT, where participants must inhibit dominant but incorrect associations to access remote semantic links. Individuals with stronger inhibitory control tend to perform better in these tasks [11, 23]. Neuroimaging studies link convergent task performance to increased activation in the dorsolateral prefrontal cortex, a region involved in top-down inhibitory control [12], aligning with the view that convergent thinking arises from the same controlled processes involved in non-creative problem solving [41].

The role of inhibition in divergent thinking is less clear. Some studies indicate that reduced inhibitory control may facilitate divergent thinking by allowing access to a wider range of loosely connected ideas [8, 35]. This reduced filtering could promote originality, consistent with associative theories of creativity whereby divergent thinking benefits from decreased executive control [2]. However, a minimal level of inhibition is still likely to be needed to maintain task engagement and prevent distraction [17]. This suggests the relationship between inhibition and creativity, at least in the context of divergent thinking tasks, may follow an inverted-U shape, where both too much and too little inhibition can impair performance, depending on the task [11]. In sum, although inhibitory control is reliably linked to convergent thinking, evidence for its role in divergent thinking is less clear.

## Cognitive flexibility in creative thinking

Cognitive flexibility, which captures the ability to shift adaptively between mental sets, perspectives, or strategies in response to changing goals or feedback [13], is also thought to be important for creativity by helping individuals move beyond habitual thought patterns [42]. This versatility allows cognitive flexibility to contribute to both convergent and divergent thinking in different ways. In convergent thinking, cognitive flexibility enables restructuring of problem representations necessary to reach a solution [43, 44]. For example, in convergent tasks like the CRAT, overcoming mental impasse caused by unhelpful initial problem framings [40] requires cognitive flexibility to disengage from ineffective strategies and reorganise mental representations to find remote or unexpected solutions [24].

In divergent thinking, cognitive flexibility seems to support the generation of ideas across multiple categories and domains, in line with the controlled attention theory of creativity, which proposes that switching between attentional states enables greater idea variety and originality [16]. However, separating executive cognitive flexibility from flexibility in divergent thinking is difficult, as the ability to produce a diverse range of idea categories is itself a core indicator of the divergent process [45]. Moreover, historical frameworks such as Guilford’s (1967) [46] suggest that flexibility in divergent thinking is a complex, non-unitary construct that can be captured through varied methodologies (cf. [47]), ranging from classic category-switching observed in divergent thinking tasks like the AUT, to more executive set-shifting as seen in the Wisconsin Card Sorting Task (WCST [48]). Based on Guildford’s [46] view, it is reasonable to expect that cognitive flexibility and flexibility in divergent thinking will be interrelated. This latter view is reinforced by studies showing that performance on set-shifting tasks, such as the WCST, predicts divergent thinking, particularly when measured in terms of flexibility and originality [19, 45]. Individuals who can shift between conceptual frameworks are potentially more likely to explore novel options and avoid fixation [19]. Despite the many studies reporting positive associations between set-shifting and divergent thinking [19, 45], some find weaker or inconsistent links, possibly due to task differences or individual variability [16, 23]. These weaker or inconsistent results further highlight the difficulty in isolating executive cognitive flexibility from divergent thinking flexibility and suggest that the link between the two may be more nuanced than Guilford [46] originally proposed.

Taken together, the role of cognitive flexibility in convergent and divergent thinking tasks aligns with dual-process models that emphasize flexible control over when to engage associative, generative processes versus evaluative processes [7]. Importantly, we note that few studies have compared the role of cognitive flexibility in convergent and divergent thinking within the same sample, limiting understanding of whether tasks that tap differentially into convergent or divergent thinking are similar or different in terms of their needs for flexible cognitive control [14]. The current study fills this gap by examining how attentional inhibition and cognitive flexibility relate to performance on convergent and divergent thinking tasks.

## Overview of the current experiments

To assess attentional inhibition in our reported studies, we calculated the interference cost of incongruent trials within a Flanker task, whereas we operationalised cognitive flexibility using two different methods. First, in Experiment 1, we focused on the switching costs associated with reversed Flanker trials to measure cognitive flexibility. Second, in Experiment 2, we expanded this approach by also including the WCST to measure set-shifting and rule-discovery. These measures of executive function (i.e., inhibition costs and flexibility costs in the Flanker task as well as perseverative errors in the WCST) were then used to predict performance across two domains of creativity: (i) divergent thinking, assessed via the AUT; and (ii) convergent thinking, measured through the CRAT.

The primary objective of this research was to investigate how attentional inhibition and cognitive flexibility may facilitate convergent and divergent thinking. We test several overarching hypotheses across our two reported studies. First, we hypothesise that stronger attentional inhibition will predict higher accuracy on the CRAT by facilitating the suppression of irrelevant associations, whereas we expect that stronger attentional inhibition will show weaker or negative correlations with originality on the AUT (Experiments 1 and 2). Second, we predict that greater cognitive flexibility, as indexed by switching costs associated with reversed flanker trials (Experiments 1 and 2) and perseverative errors in the WCST (Experiment 2), will positively relate to both CRAT and AUT performance. Specifically, we expect cognitive flexibility to exert a particularly strong influence on the originality and flexibility of divergent ideas (Experiments 1 and 2). Finally, we explore whether these measures of executive function (i.e., attentional inhibition and cognitive flexibility) influence convergent thinking by shaping the specific problem-solving strategies (i.e., insight-based vs. analytic) that individuals employ on the CRAT (Experiment 2).

### Experiment 1

To reiterate, prior research has demonstrated that both attentional inhibition and cognitive flexibility contribute to creative cognition [7, 8]. However, inconsistencies remain regarding their distinct roles in supporting convergent versus divergent thinking. The present experiment addresses this by employing a modified flanker task [49], designed to provide trial-level behavioural indices of attentional inhibition and cognitive flexibility through three trial types: congruent, incongruent, and reversed. Across trials, participants were asked to respond with a key button press that aligned with the direction of a central arrow stimulus. In congruent trials, the central arrow was surrounded by flanking arrows (flankers) pointing in the same direction, requiring no inhibition. These trials served as a baseline measure of performance. In incongruent trials, the flankers pointed in the opposite direction, generating conflict that necessitated the suppression of irrelevant stimuli, thereby engaging attentional inhibition. Reversed trials constituted a novel modification in which participants had to respond to the central arrow’s direction with the *opposite* button press, requiring both response inhibition and rule switching. This manipulation taps into cognitive flexibility through the need to adapt to changing task rules.

In the study we report, reaction time (RT) and accuracy differences between congruent and incongruent trials, commonly referred to as the “congruency effect” [50], provided well-established indices of attentional inhibition, reflecting the ability to suppress distracting information. In contrast, RT and accuracy differences between reversed and congruent trials provided indices of cognitive flexibility, reflecting the capacity to adapt to shifting task demands and switch response sets [51]. Full details on task parameters, timing, response mappings, and visual stimuli are provided in the Methods section below.

Creativity was assessed using two established tasks chosen to capture convergent and divergent thinking. The CRAT [22, 52] was employed to assess convergent thinking, requiring participants to find a single solution word that semantically links three cue words, engaging controlled semantic retrieval, focused attention, and constraint satisfaction. The CRAT captures the ability to narrow down and integrate information to identify a single correct solution, which, as noted earlier, appears to rely on executive control processes such as attentional inhibition to inhibit incorrect or irrelevant associations, and cognitive flexibility to shift between idea generation and idea evaluation to identify the singular correct answer. The AUT [20, 26] measures divergent thinking by requiring participants to generate multiple, novel uses for everyday objects. The AUT was employed to capture key aspects of divergent thinking, such as fluency (number of responses), flexibility (range of responses), and originality (uniqueness of responses), which reflect the capacity to produce a broad range of ideas. This task is widely used due to its ecological validity and its sensitivity to executive processes involved in associative idea generation and exploration.

By combining behavioural indices of inhibition and flexibility from the flanker task with independent measures of convergent and divergent thinking, this experiment aims to clarify how attentional inhibition and cognitive flexibility independently support different forms of creative cognition. Based on the available literature, we hypothesised the following:

- Hypothesis 1: Stronger attentional inhibition, indexed by reduced flanker congruency costs (i.e., smaller accuracy and RT differences between incongruent and congruent trials), will predict higher accuracy and solution rates on the CRAT, reflecting effective suppression of irrelevant semantic associations during convergent thinking [11, 23].
- Hypothesis 2: Attentional inhibition will show weaker or negative correlations with AUT performance (fluency, originality, flexibility), consistent with evidence that reduced inhibition facilitates broader ideational breadth and flexibility during divergent thinking [35].
- Hypothesis 3: Greater cognitive flexibility, indexed by smaller switch costs (i.e., accuracy and RT differences between reversed and congruent trials), will positively relate to both CRAT and AUT performance, with a stronger effect expected for the AUT because of the necessity of set-shifting and adaptive thinking in generating diverse ideas [16, 49].

### Methodology

#### Participants

Participants were 55 undergraduate and postgraduate students aged between 18 and 56 years (*M*_*age*_ = 24.2 years, *SD*_*age*_ = 8.21) at the University of Lancashire. The participants were mainly female (*n* = 41) and right-handed (*n* = 45). The sample included 41 White British (74.6%), 8 Asian (14.6%), 2 Black British (3.6%), 2 Russian (3.6%), 1 Indian (1.8%) and 1 African (1.8%). All participants reported normal to corrected-to-normal vision and hearing and were fluent in English and proficient in reading and writing. Participants gave written consent and were awarded eight course credits, where relevant, and a £10 Amazon voucher incentive as compensation for their time.

A power calculation was performed to determine the sample size using PANGEA [53], as this tool is specifically designed to handle the hierarchical structure of mixed-model designs [53]. The calculation determined that a sample of 52 participants was required with power set at 0.8 and alpha set at 0.05, based on expectations of a medium effect size *(d* = 0.5), commensurate with prior research [54]. A sample of 55 participants was therefore recruited to allow for attrition. After completing the study, we also checked the adequacy of this sample size by conducting a sensitivity power analysis using Fisher-*z* calculations. This confirmed that our sample of 55 participants achieved a power of 0.82 to detect a medium effect size (*r* = .38), which we deemed to be adequate based on prior work linking attentional control to creative thinking [16, 19, 49].

#### Materials

##### Flanker task

 The flanker task was presented using E-Prime (Version 2.0.10; Psychology Software Tools, Pittsburgh, PA) and displayed on a 14-inch LCD screen in 30-point Times New Roman font as black text on a white background. At 55 cm viewing distance, each character subtended approximately 1^◦^ of visual angle and represented a normal size for reading.

Attentional inhibition and cognitive flexibility were assessed using a modified version of the flanker task [48], which was adapted from our previous protocol [55]. The task involved a sequence of trials, with each trial requiring participants to respond with a key button-press to indicate the direction of a centrally presented arrow that was flanked by two arrows to its left and two arrows to its right. The task consisted of 438 such trials, evenly split into congruent, incongruent, and reversed configurations (146 of each), with each trial type including an equal number of left-pointing and right-pointing target arrows. On congruent trials, all five arrows were presented in green and pointed in the same direction. On incongruent trials, all five arrows were also presented in green, but the central arrow pointed in the opposite direction to the flankers, generating conflict and requiring inhibitory control. Reversed trials presented red arrows, signalling to participants that they should deliberately respond in the opposite direction to the central arrow, thus overriding the usual stimulus–response mapping. We did not further subdivide reversed trials by congruency, because four out of five arrows were aligned in direction in both cases, making perceptual differences negligible [55]. To verify this assumption, we compared reversed incongruent and reversed congruent trials and found no significant differences in accuracy or reaction times, supporting our decision to collapse across congruency in reversed trials.

**Fig. 1:**
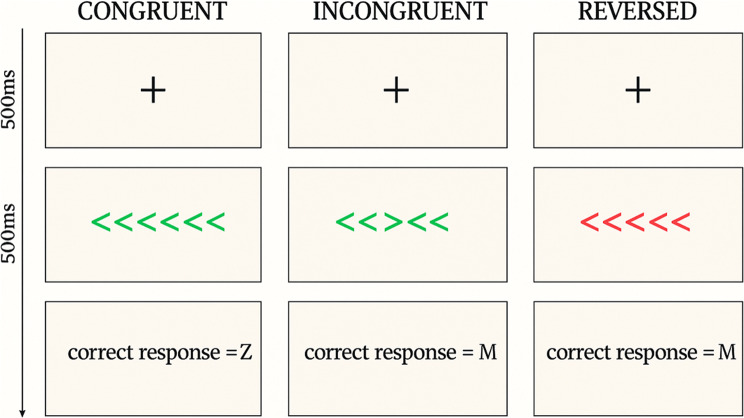
Graphical representations of the congruent, incongruent and reversed flanker trials with correct responses

Responses were collected via standard keyboards, with participants using the “Z” key for left responses and the “M” key for right responses. On congruent and incongruent (green) trials, key presses corresponded to the central arrow’s direction, whereas on reversed (red) trials, participants responded with the opposite key (e.g., “M” for a left-pointing arrow). Figure 1 shows the trial set-up, with correct responses also displayed. Each trial began with a fixation cross (500 ms), followed by the presentation of the stimulus up to a maximum response duration of 500 ms, with an inter-trial interval (blank screen) of 500 ms. At the beginning of the task, to ensure comprehension all participants completed six practice trials (two per trial type) with feedback also being given. The full set of experimental trials was presented in a single block to maintain attentional demand.

##### Compound remote associates test 

A set of 16 CRAT items were selected from Bowden and Jung-Beeman [22] to measure convergent thinking. It is typical to divide CRAT items into easy and difficult sets [56], hence selection of items included an even split of 8 easy and 8 difficult problems, based on normative data from Bowden and Jung-Beeman [22]. Although not a primary goal of the study, we considered it important to ensure an even distribution of difficulty levels, since there are potential differences in solving processes between easy and difficult items [56]. Each CRAT item consisted of the presentation of three single cue words, with the participant being asked to find a fourth word that combined with each of the three presented words to make a common word or phrase.

The cue words for each CRAT item were presented using Qualtrics software and were displayed on a 14-inch LCD screen in 30-point Times New Roman font as black text on a white background. Responses were recorded via the computer’s keyboard and mouse. Participants had 30 s to solve each item and were asked to type their response into a text box. This 30 s response window was selected to ensure that participants had sufficient opportunity to attempt each item and to reduce the likelihood of floor effects, particularly given the inclusion of difficult problems. By providing a longer window than some previous studies (e.g., 15–20 s [57]), we aimed to maintain adequate variability in performance, which is essential for examining the relationships between individual differences in convergent thinking and attentional control measures. If participants wanted to move on before the timer had finished, they could click “Next” using the mouse. Participants were instructed to write “DK” if they could not think of a solution. The procedure was explained to participants at the start of the experiment, and they had one practice CRAT item before starting the main experiment.

##### Alternative uses task 

Six AUT problems were selected to measure divergent thinking. The participants were instructed that they would see a written word on the top of a blank page, and that they would be requested to think of as many different and unusual uses for that object as possible and needed to write their answers on the blank page. They were told that they had 2 min to think of as many possible uses, and after the timer alarmed, they should stop writing immediately and move onto the next problem. After the instructions were explained to the participant, they were shown one example by the experimenter. This example showed the word *brick* and included five possible answers: *a doorstop*,* a paper weight*,* a step*,* a diving aid* and *to break a window.* Each participant was then presented the test stimuli, one by one in a randomised sequence, and asked to generate as many possible uses as possible for each stimulus before the timer expired.

Although there are numerous ways to score the AUT (see [58]), we employed the traditional scoring dimensions established by Guilford [46] and later standardised by Torrance [59]. Specifically, we quantified divergent thinking through three dimensions: fluency, originality and flexibility [25]. AUT fluency was the overall sum of generated uses per test averaged across the six AUTs. For AUT originality, each answer that was unique to a participant (i.e., no other participants thought of that use) was coded as 1, while any answer provided by more than one participant was coded as 0. The final AUT originality score was the sum of original answers per test averaged across the six AUTs. Third, we scored participants’ AUT flexibility to reflect the range of idea categories for each item. The AUT flexibility score was the overall sum of idea categories per test averaged across the six AUTs.

#### Procedure

Each participant provided written informed consent prior to taking part in the experiment. Participants completed the flanker task on a laptop computer and were provided with instructions and completed practice trials to ensure familiarity with the task and the computer. Participants then completed the CRAT items via Qualtrics, and the AUT items using pen and paper. The flanker task, CRAT and AUT were counterbalanced across participants. In total, the experiment took approximately 40 min to complete. On completion, each participant received a written debrief. Ethical approval was obtained from the University of Lancashire Science Ethics Committee (approval code: PSYSOC463_Amendment_20_May_2024).

## Results

All analyses were conducted using *R* version 4.3.2 [60]. Data cleaning was performed with *dplyr* (1.1.4) and *tidyr* (1.3.0). Linear and generalised linear mixed-effects models were fitted using lme4 (1.1–35.1). Multicollinearity diagnostics employed car (3.1-2), and mediation analyses used mediation (4.5.0). Additionally, *readxl* (1.4.3) was used for importing raw data files. All linear models were fitted using base *R* functions.

Mean accuracy scores for congruent, incongruent and reversed trials were obtained by averaging the proportion of correct responses across participants for each trial type. Descriptive statistics for mean accuracy by trial type are presented in Table 1.

**Table 1:** Mean accuracy (proportion of correct responses) by trial type (congruent, incongruent, reversed)

Trial Type	Mean RT (ms)	SD RT (ms)
Congruent	441.9	154.34
Incongruent	479.0	132.11
Reversed	491.0	161.12

### Accuracy

To examine the effect of trial type on accuracy, we fitted a Generalised Linear Mixed Model (GLMM) with a binomial link function, with trial type entered as a fixed effect and with by-participant random intercepts and slopes. The model revealed a significant main effect of trial type. Compared to congruent trials (estimated marginal accuracy = 96.0%), participants were significantly less accurate on incongruent trials (estimated difference = − 3.8%, *p* < .001) as well as on reversed trials (estimated difference = − 9.6%, *p* < .001), reflecting greater difficulty with increased cognitive conflict (see Table 2).

**Table 2:** Fixed effect estimates (log-odds scale) for accuracy by trial type (congruent, incongruent, reversed)

Predictor	Estimate (log-odds)	SE	z	*p*	Cohen’s d
Intercept (congruent)	3.189	0.256	12.46	< 0.001 ***	—
Incongruent	–0.633	0.146	–4.34	< 0.001 ***	–0.35
Reversed	–1.370	0.154	–8.89	< 0.001 ***	–0.75

Estimates are on the log-odds scale. Congruent trials served as the reference level. Cohen’s *d* values were approximated from the log-odds estimates [61]. Estimated marginal means: congruent = 96.0%, incongruent = 92.8%, reversed = 86.0%

In addition to fixed effects, participant-level random effects (intercepts and trial type slopes) were extracted to capture individual variation in accuracy across conditions, expressed as deviations in log odds from the population average (Table 3). These values are not raw percentages and should be interpreted cautiously.

**Table 3:** Participant-level random effect estimates (log-odds scale) for accuracy by trial type (congruent, incongruent, reversed)

Measure	Mean	SD	Min	Max
Intercept (Congruent)	–0.018	1.406	–4.585	1.730
Incongruent (Slope)	0.061	0.684	–2.281	1.773
Reversed (Slope)	0.008	1.500	–2.144	6.333

Values represent participant-level random effect estimates from the binomial GLMM. These reflect deviations in log odds from the population-level fixed effects and are not raw accuracy percentages

Four separate linear regression models were used to examine whether accuracy in the flanker task predicted performance on creative thinking tasks: CRAT accuracy, AUT fluency, AUT originality and AUT flexibility. Predictors included accuracy scores in the baseline (congruent), incongruent, and reversed trials. Model 1 was not significant, *F*(3,51) = 2.46, *p* = .073, *R*² = 0.1 (Adjusted *R*²), but individual predictors revealed that higher accuracy on incongruent trials significantly predicted higher CRAT scores, *b* = 0.106, *p* = .031. Neither baseline nor reversed trial accuracy were significant predictors. Model 2 was not significant, *F*(3, 51) = 0.18, *p* = .908, *R*² = 0.011, (Adjusted *R*² = − 0.048) and showed that no flanker condition significantly predicted AUT fluency scores. Model 3 was significant, *F*(3, 51) = 3.24, *p* = .030*, *R*² = 0.16 (Adjusted *R*² = 0.11) and revealed that incongruent accuracy significantly predicted AUT originality scores (*b* = 1.77, *p* = .013), while baseline and reversed accuracy did not. Model 4 was not significant, *F*(3, 51) = 0.97, *p* = .41, *R*² = 0.054 (Adjusted *R*² = 0.018), showing that no flanker condition predicted AUT flexibility. The full model results are shown in Table 4.

**Table 4:** Linear regression models predicting CRAT accuracy, AUT fluency, AUT originality and AUT flexibility outcomes from flanker task accuracy

Outcome	Predictor	B	SE	t	*p*
Model 1: CRATs	Intercept	0.603	0.028	21.32	< 0.001 ***
	Accuracy (congruent; baseline)	–0.044	0.030	–1.45	0.153
	Accuracy (incongruent)	0.106	0.048	2.22	0.031 *
	Accuracy (reversed)	–0.039	0.031	–1.26	0.213
Model 2: AUT fluency	Intercept	4.518	0.262	17.21	< 0.001 ***
	Accuracy (congruent; baseline)	–0.012	0.279	–0.05	0.965
	Accuracy (incongruent)	0.245	0.445	0.55	0.585
	Accuracy (reversed)	0.022	0.284	0.08	0.938
Model 3: AUT originality	Intercept	3.650	0.406	8.998	< 0.001 ***
	Accuracy (congruent; baseline)	–0.414	0.432	–0.96	0.343
	Accuracy (incongruent)	1.767	0.688	2.57	0.013 *
	Accuracy (reversed)	–0.263	0.440	–0.60	0.552
Model 4: AUT flexibility	Intercept	4.422	0.310	14.272	< 0.001***
	Accuracy (congruent; baseline)	–0.003	0.005	–0.656	0.515
	Accuracy (incongruent)	–0.023	0.035	–0.661	0.538
	Accuracy (reversed)	–0.051	0.035	–1.474	0.147

*p* < .05 *, *p* < .01 **, *p* < .001 ***

### Reaction time

RTs in the flanker task were filtered to exclude implausibly fast or slow responses (< 200 ms or > 3000 ms), consistent with recommended practice for RT cleaning in cognitive control tasks [62]. After trimming, a total of 23,180 trials were retained for analysis, comprising 8,102 congruent trials, 7,522 incongruent trials, and 7,556 reversed trials. Descriptive statistics for mean RT by trial type are presented in Table 5.

**Table 5:** Mean RT (ms) by trial type (congruent, incongruent, reversed)

Trial Type	Mean RT (ms)	SD RT (ms)
Congruent	441.9	154.34
Incongruent	479.0	132.11
Reversed	491.0	161.12

A linear mixed-effects model was used to examine the effect of trial type on RT, with trial type entered as a fixed effect and with by-participant random intercepts and slopes. The model revealed a significant main effect of trial type (see Table 6). To facilitate interpretation of the reaction-time effects, standardized mean differences (Cohen’s *d*) were calculated by dividing the mean differences by the pooled standard deviation across conditions. Compared to congruent trials (*M* = 441.87 ms), responses were significantly slower for incongruent trials (*M* difference = 38.81 ms, *p* < .001) and for reversed trials (*M* difference = 48.70 ms, *p* < .001), consistent with increased cognitive demands.

**Table 6:** Fixed effects estimates for RT (ms) by trial type (congruent, incongruent, reversed)

Trial type	Estimate	SE	t	*p*	Cohen’s d
Intercept (congruent)	441.87	8.02	55.07	< 0.001 ***	—
Incongruent	38.81	2.73	14.21	< 0.001 ***	0.27
Reversed	48.70	2.99	16.26	< 0.001 ***	0.31

Estimates for incongruent and reversed trials represent mean differences in RT (ms) relative to the congruent condition. Cohen’s *d* values were calculated by dividing the mean differences by the pooled standard deviation across conditions

Random intercepts and slopes for each participant were extracted to capture individual attentional profiles. Three subject-level metrics were derived: (i) SustainedRT, reflecting baseline RT on congruent trials (random intercept); (ii) InhibitionCost, the RT change for incongruent minus congruent trials; and (iii) FlexibilityCost, the RT change for reversed minus congruent trials. A more positive score on the latter two metrics would therefore represent a larger cost (i.e., poorer performance on incongruent or reversed trials relative to congruent trials). These metrics were used as predictors in subsequent regression models.

A multiple linear regression model examined whether SustainedRT, InhibitionCost, and FlexibilityCost predicted CRAT accuracy (Model 1), AUT fluency (Model 2), AUT originality (Model 3) and AUT flexibility (Model 4). Model results are presented in Table 7.

**Table 7:** Linear regression models predicting CRAT accuracy, AUT fluency, AUT originality and AUT flexibility outcomes from flanker RT metrics

Model	Predictor	B	SE	β	t	*p*
Model 1: CRAT accuracy	(Intercept)	0.660	0.020	—	32.41	< 0.001***
	SustainedRT	0.0004	0.0003	0.13	1.17	0.25
	InhibitionCost	–0.0048	0.0023	–0.28	–2.04	0.046 *
	FlexibilityCost	–0.0076	0.0023	–0.38	–3.31	0.002 **
Model 2: AUT fluency	(Intercept)	4.56	0.27	—	16.84	< 0.001***
	SustainedRT	–0.0048	0.0046	–0.14	–1.04	0.31
	InhibitionCost	0.0042	0.0310	0.02	0.13	0.89
	FlexibilityCost	–0.0097	0.0304	–0.04	–0.32	0.75
Model 3: AUT originality	(Intercept)	4.42	0.31	—	14.27	< 0.001***
	SustainedRT	–0.0034	0.0053	–0.08	–0.66	0.51
	InhibitionCost	–0.123	0.0355	–0.50	–3.46	0.001 **
	FlexibilityCost	–0.0512	0.0348	–0.21	–1.47	0.15
Model 4: AUT flexibility	(Intercept)	2.618	0.148	—	17.64	< 0.001***
	SustainedRT	–0.001	0.003	–0.78	–0.55	0.582
	InhibitionCost	0.022	0.017	0.287	1.27	0.211
	FlexibilityCost	–0.018	0.017	–0.243	–1.06	0.293

Model 1 (predicting CRAT accuracy) was significant, *F*(3, 51) = 23.69, *p* < .001, explaining 56% of the variance in CRAT scores (Adjusted *R*² = 0.56). InhibitionCost and FlexibilityCost were both significant negative predictors of CRAT accuracy. Participants who exhibited greater RT costs for incongruent and reversed trials tended to score lower on the CRAT, indicating that poorer inhibitory control and reduced cognitive flexibility were associated with lower CRAT scores. SustainedRT was not a significant predictor. Model 2 (predicting AUT fluency) was not statistically significant, *F*(3, 51) = 0.48, *p* = .70, Adjusted *R*² = − 0.03, and none of the predictors reached significance. Model 3 (predicting AUT originality) was significant, *F*(3, 51) = 20.59, *p* < .001, explaining 52% of the variance (Adjusted *R*² = 0.52). InhibitionCost emerged as a significant negative predictor of AUT originality scores (*p* = .001), while SustainedRT and FlexibilityCost were not significant. Model 4 (predicting AUT flexibility) was not statistically significant, *F*(3, 51) = 0.76, *p* = .52, *R*² = 0.043, Adjusted *R*² = –0.013, and none of the predictors reached significance.

## Discussion

Consistent with Hypothesis 1, greater accuracy on incongruent trials predicted higher CRAT scores, highlighting the importance of attentional inhibition in convergent thinking. This relationship was further supported by RT data, where lower inhibition costs between congruent and incongruent flanker trials – indicating better attentional inhibition – were significantly associated with higher CRAT scores (i.e., enhanced convergent thinking).

Hypothesis 2, which predicted a weaker or absent relationship between attentional inhibition and divergent thinking, was partially confirmed. Increased accuracy on incongruent trials, reflecting more efficient attentional inhibition, did not predict AUT fluency or AUT flexibility. However, it was seen to predict AUT originality. Similarly, although inhibition costs did not predict AUT fluency or AUT flexibility, it was found to be a significant negative predictor of AUT originality. These results suggest that inhibitory control may only be relevant for the originality of idea generation in this divergent thinking task.

Hypothesis 3 was also partially supported. Cognitive flexibility, as indexed through accuracy on reversed trials, neither predicted CRAT accuracy nor AUT scores (fluency, originality or flexibility). However, when cognitive flexibility was indexed in terms of RT costs on reversed trials, it did significantly predict CRAT accuracy but was not significantly relate to any of the AUT scores. These results suggest that cognitive flexibility (when derived as an RT cost metric) supports the generation of successful solutions to CRAT items (i.e., enhanced convergent thinking), but is not associated with divergent thinking in the AUT.

### Experiment 2

Experiment 1 demonstrated that individual differences in attentional inhibition and cognitive flexibility predicted performance on a convergent thinking task (i.e., the CRAT). Specifically, participants who experienced lower inhibition and flexibility costs – reflecting more efficient attentional control – achieved higher accuracy on CRAT items. We note that the way in which people solve CRAT problems has become a topic of increasing interest in the creativity field [63, 64], with discussion centring on whether problems are solved via an analytic process or via an insight process. An analytic process generally involves deliberate, step-by-step reasoning and focused search within a problem space [65]. In contrast, insight is characterised by sudden restructuring, constraint relaxation, and the spontaneous emergence of a solution, giving rise to an “*Aha!*” experience [57].

Based on this latter distinction concerning the strategies that are deployed to solve CRAT items, the results from Experiment 1 could be explained in two ways. First, if CRATs are solved via an analytic approach, then efficient attentional inhibition may help to suppress irrelevant or misleading information, while cognitive flexibility may support an improved ability to shift between rules or perspectives, allowing for repeated generation and evaluation of ideas. Second, if problems are instead solved via insight, then efficient inhibition may allow for disengagement from ineffective analytic efforts and the reorientation of attention toward more remote associations, with flexibility facilitating the shift in perspective needed for sudden restructuring processes that underpin the emergence of insight. To distinguish between these two alternative accounts, in Experiment 2 we employed a trial-level self-report measure of solving strategy that allowed us to classify solution responses in the CRAT as being based on either analytic or insight processes. In this way, we were able to examine directly whether the benefits of improved attentional inhibition and cognitive flexibility on CRAT performance are mediated by solving strategy.

In contrast, the findings from Experiment 1 for divergent thinking (measured via the AUT) were less consistent than they were for convergent thinking (measured via the CRAT). Attentional inhibition showed a modest relationship with AUT originality, but not AUT fluency or AUT flexibility. Furthermore, cognitive flexibility did not significantly predict any AUT dimension. These results partially support the notion that attentional inhibition and cognitive flexibility contribute more strongly to convergent than divergent thinking and suggest that divergent thinking tasks may be less dependent on executive control processes (e.g., evaluation or attentional shifting), and more dependent on associative fluency and idea generation.

We acknowledge that although the reversed flanker trials in Experiment 1 provided a fine-grained, trial-level index of cognitive flexibility, they may nevertheless have only captured momentary attentional shifts. This possibility suggests that it would be valuable to explore this topic using a broader, sustained set-shifting ability, as measured using tasks like the WCST [48]. Consequently, in Experiment 2, we included the WCST, a well-validated behavioural task that measures cognitive flexibility as the ability to shift strategies in response to changing rules (full task information can be found in the Method). By including this additional measure, we were able to examine whether more general cognitive flexibility capacities explained unique variance in convergent (CRAT) and divergent (AUT) thinking that were not identified in Experiment 1.

Our first two hypotheses for Experiment 2 aimed to replicate the results reported in Experiment 1, with subsequent hypotheses being drawn from the available literature outlined above:

- Hypothesis 1: Participants with lower flanker-based attentional inhibition and cognitive flexibility costs will perform better on the CRAT.
- Hypothesis 2: Attentional inhibition costs will negatively predict originality scores on the AUT, but not AUT fluency or AUT flexibility.
- Hypothesis 3: Better performance on the WCST, reflecting broader cognitive flexibility, will predict higher CRAT accuracy, further supporting findings from Experiment 1.
- Hypothesis 4: Performance on the WCST will positively predict AUT originality and AUT flexibility, but not AUT fluency, suggesting that broader cognitive flexibility as measured using the WCST, may support the originality component of divergent thinking and the flexibility of ideas, even if not directly influencing idea fluency.
- Hypothesis 5: Problem-solving strategy (analytic vs. insight) will be explored as a predictor of CRAT accuracy and will additionally be examined as a potential mediator of the relationship between our attentional metrics (inhibition and cognitive flexibility) and CRAT accuracy.

## Method

### Participants

Participants were 49 undergraduate and postgraduate students aged between 18 and 48 years (35 females, 12 males, and 2 preferred not to say; *M*_*age*_ = 27.5 years, *SD*_*age*_ = 8.17) at the University of Lancashire. The sample included 40 White British (81.6%), 6 Asian (12.2%), 1 Black British (2%), 1 Lithuanian (2%) and 1 Russian (2%). A sensitivity power analysis using Fisher-*z* calculations confirmed that this sample achieved 0.77 power to detect a moderate to large effect (*r* ≈ .48; [66]), which is suitable given the effect sizes reported in Experiment 1. All participants reported normal to corrected-to-normal vision and hearing, were fluent in English and proficient in reading and writing. Participants gave written consent and were awarded 10 course credits, where relevant, and a £10 Amazon voucher incentive as compensation for their time.

### Materials

Experiment 2 builds upon Experiment 1 by employing the same design to determine the role of attentional inhibition and cognitive flexibility in two creativity tasks that tapped into either convergent or divergent thinking. As in Experiment 1, we derived metrics of attentional inhibition and cognitive flexibility using a modified version of the flanker task. The CRAT was utilised in the same way as Experiment 1, but this time with an additional measure of solving strategy (described below). The AUT was utilised and scored in the same way as Experiment 1 to provide scores for AUT fluency, AUT originality and AUT flexibility.

#### CRAT solving strategy 

To examine individual solving strategy (i.e., analytic vs. insight) in the CRAT, participants were asked to select how they solved each CRAT item by selecting one of four multi-choice answers (taken from [38]):

(i) Complete Strategy: when you thought of the word, at first you did not know whether it was the answer, but after thinking about it strategically (e.g., trying to combine the single word with each of the three problem words) you figured out that it was the answer.
(ii) Partial Strategy: you did not immediately know the word was the answer, but you did not have to think about it much either (e.g., after figuring out how the solution went with the first two stimulus words, you realised that it was the solution).
(iii) Partial Insight: you had a weaker feeling of insight (not as strong as a rating of 4; e.g., you felt that the word you thought of might have been the answer, but it was not as obvious as “*Of course!*”). You may have had to check the solution with one of the words to make sure it was correct.
(iv) Complete Insight: as soon as you thought of the word you knew that it was the answer; the solution word came with a feeling that it was correct (e.g., “*it popped into my head*”; “*of course!*”; “*I had an Aha!*”).

#### Wisconsin card sorting test (WCST)

**Fig. 2:**
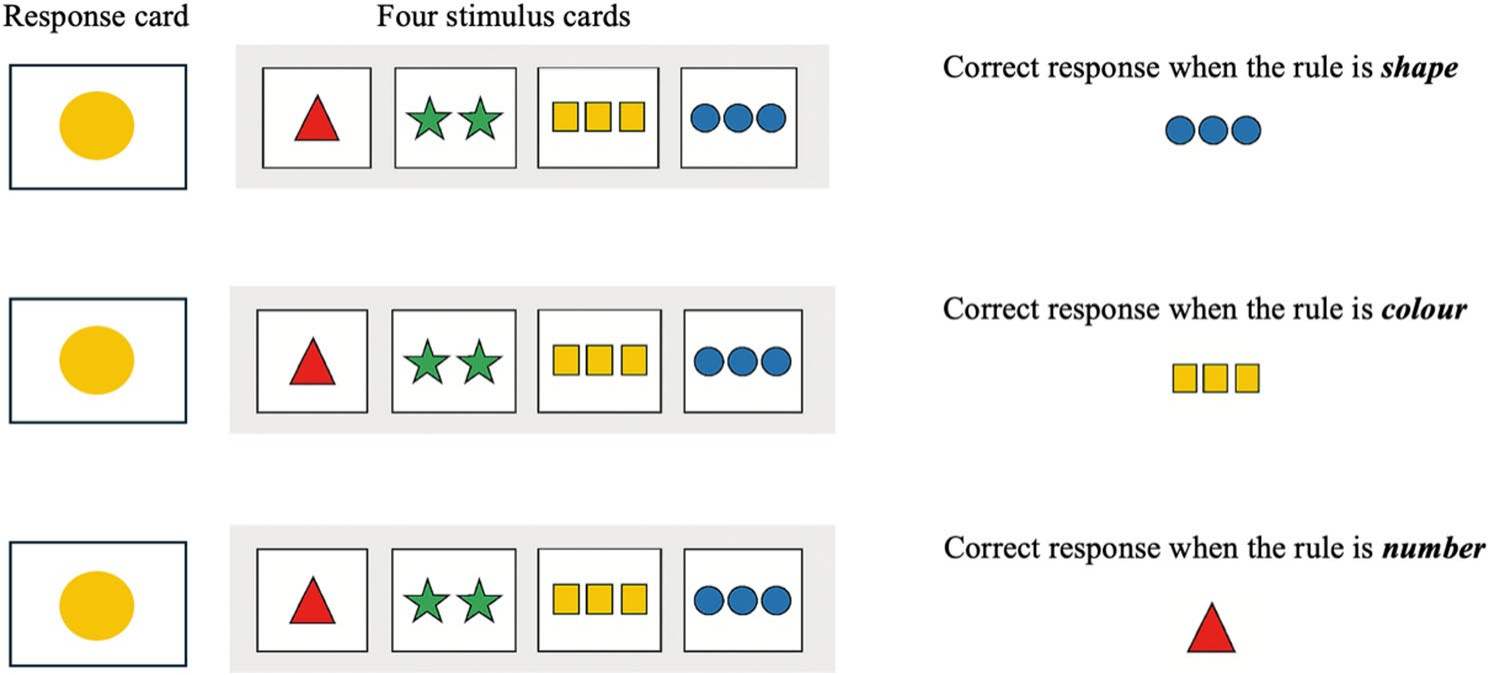
A visual representation of each correct response to the corresponding sorting rule in the Wisconsin card sorting task

To introduce a broader measure of cognitive flexibility than cost metrics derived from the flanker task, we used the WCST to measure how well participants could adapt their responses to changes in contingencies [48]. In our computerised version of the WCST, each participant was shown four stimulus cards on a computer screen, respectively depicting one red triangle, two green stars, three yellow squares, and four blue circles. The participants were then asked to sort a series of response cards according to one of the following rules: (i) the colour of the figures on the stimulus cards; (ii) the number of figures on the stimulus cards; and (iii) the form of the figures on the stimulus cards (see Fig. 2). The participants were given one response card at a time and were instructed to respond using keys on a keyboard to indicate which stimulus set the response card aligned to, with no set time limit.

After each sorting trial, participants received feedback on the computer screen as to whether the sort was correct or incorrect. When the participant made 10 consecutive correct sorts, the sorting rule changed, without warning. The challenge for the participants in this task was therefore to adapt their responses as the sorting rule suddenly changed. The fewer trials a participant needed to adapt their response after a rule switch, the more flexible a person was in adapting their response to the new contingency. In the WCST, the index that captures this latter type of cognitive flexibility is referred to as being a measure of “perseverative error”, which captures how often a participant continues to sort cards after an outdated and incorrect sorting rule [47]. A person who is flexible in their way of responding will demonstrate a lower number of perseverative errors, and faster RTs, because they will be more efficient in noticing and adapting to the new sorting rule. The sorting rule in the current WCST was randomly generated, with the exception that the same sorting rule could not repeat two times in a row (e.g., *colour* followed by *colour*). The rule-sequence repeated until the participant had completed six sorting categories or 128 trials.

### Procedure

Each participant provided written informed consent prior to taking part in the experiment. Participants completed the flanker task, the CRAT and AUT, which were all identical to Experiment 1, except for a solving strategy response being requested after each CRAT item. The WCST was presented on PsychoPy 3.0, with participants being provided with instructions and practice trials to ensure familiarity before completing the main tasks. All tasks were counterbalanced across participants. In total, the experiment took approximately 50 min and on completion, each participant received a written debrief. Ethical approval was obtained from the University of Lancashire Science Ethics Committee (approval code: PSYSOC 463 Amendment_20May2024).

## Results

### Flanker accuracy

Descriptive statistics for mean accuracy by trial type in the flanker task are presented in Table 8.

**Table 8:** Mean accuracy (proportion of correct responses) by trial type (congruent, incongruent, reversed)

Trial type	Mean Accuracy	SD Accuracy
Congruent	0.854	0.02
Incongruent	0.777	0.03
Reversed	0.656	0.03

As in Experiment 1, we examined the effect of trial type on accuracy by fitting a GLMM with a binomial link function, with trial type entered as a fixed effect and with by-participant random intercepts and slopes. The model revealed a significant main effect of trial type (*p* < .001). Participants were most accurate on congruent trials (*M* = 0.85), followed by incongruent (*M* = 0.78) and reversed trials (*M* = 0.66). Both incongruent and reversed trials showed significantly lower accuracy than congruent trials (both *p*s < 0.001; see Table 9).

**Table 9:** Fixed effect estimates (log-odds scale) for accuracy by trial type (congruent, incongruent, reversed)

Predictor	Estimate (log-odds)	SE	z	*p*	Cohen’s d
Intercept (Congruent)	1.770	0.136	13.00	< 0.001 ***	—
Incongruent	−0.516	0.095	−5.41	< 0.001 ***	−0.28
Reversed	−1.120	0.098	−11.40	< 0.001 ***	−0.62

Estimates are on the log-odds scale. Congruent trials served as the reference level. Cohen’s d values were approximated from the log-odds estimates [61]. Estimated marginal means: congruent = 85.4%, incongruent = 77.7%, reversed = 65.6%.

Participant-level random-effects estimates (intercepts and trial-type slopes) were extracted from the GLMM to quantify individual differences in baseline accuracy across trial types. These estimates represent deviations (in log odds) from the population-level fixed effects. Accuracy was highest in the congruent condition and significantly decreased in both the incongruent (estimate = − 0.51, *p* < .001, *d =* − 0*.28*) and reversed (estimate = − 1.09, *p* < .001, *d* = − 0.60) conditions, indicating greater difficulty with conflicting and reversed stimuli. Random effects indicated substantial between-participant variability in baseline accuracy across trial type (see Table 10).

**Table 10:** Participant-level random-effects estimates (log-odds scale) for accuracy by trial type (congruent, incongruent, reversed)

Measure	Mean	SD	Min	Max
Intercept (Congruent)	−0.014	0.927	−3.087	1.720
Incongruent (Slope)	0.004	0.469	−1.570	0.992
Reversed (Slope)	0.009	0.521	−1.213	1.244

Values represent participant-level random-effect estimates from the binomial GLMM. These reflect deviations (log odds) from population-level fixed effects and are not raw accuracy percentages

### Flanker reaction time

RTs in the flanker task were filtered to exclude implausibly fast or slow responses (< 200 ms or > 3000 ms). After trimming, 20,508 trials were retained for analysis, comprising 7,156 congruent, 6,695 incongruent, and 6,657 reversed trials. Descriptive statistics for RT by trial type are presented in Table 11.

**Table 11:** Mean RT (ms) by trial type (congruent, incongruent, reversed)

Trial type	Mean RT (ms)		SD RT (ms)
Congruent		436.0	122.0
Incongruent		470.0	123.0
Reversed		488.0	126.0

An LMM was used to examine the effect of trial type on RT with trial type entered as a fixed effect and by-participant random intercepts and slopes. The model revealed a significant main effect of trial type. Compared with congruent trials (*M* = 436.01 ms), participants responded significantly slower on incongruent trials (mean difference = 34.65 ms, *p* < .001) and reversed trials (mean difference = 52.67 ms, *p* < .001), indicating robust attentional inhibition and flexibility costs (see Table 12).

**Table 12:** Fixed effects estimates for RT (ms) by trial type (congruent, incongruent, reversed)

Trial type	Estimate (ms)	SE	t	*p*	Cohen’s d
Intercept (Congruent)	436.01	10.37	42.06	< 0.001	—
Incongruent	34.65	3.01	11.50	< 0.001	0.28
Reversed	52.67	5.00	10.53	< 0.001	0.42

Estimates for incongruent and reversed trials represent mean differences in RT (ms) relative to the congruent condition. Cohen’s *d* values were calculated by dividing the mean differences by the pooled standard deviation across conditions

Participant-level random effects were extracted from this model to characterise individual differences on congruent, incongruent and reversed trials. Descriptive statistics for these random effects are shown in Table 13.

**Table 13:** Participant-level random-effect estimates for RT by trial type (congruent, incongruent, reversed)

Measure	Mean	SD	Min	Max
Intercept (Congruent)	0.003	65.83	−185.01	171.44
Incongruent (Slope)	0.002	12.42	−44.10	21.25
Reversed (Slope)	0.009	24.78	−64.52	51.72

Values represent participant-level random-effect estimates from the LMM

### Perseverative error and reaction time on the Wisconsin card sorting task

Performance on the WCST was summarised at the participant level using perseverative error scores and log-transformed RTs to reduce skewness in the RT data distribution (see Table 14). Perseverative error is interpreted as an inverse measure of cognitive flexibility, where higher WCST perseverative error reflects lower cognitive flexibility. WCST RTs were also interpreted as an inverse measure of cognitive flexibility, where higher RTs reflect lower cognitive flexibility.

**Table 14:** Descriptive data for the WCST performance variables (RT and perseverative error), with RT presented in both raw (ms) and log-transformed forms

Variable	M	SD	Min	Max
WCST RT	2260.25	831.56	1212.60	4848.27
WCST RT (log transformed)	0.51	2.03	–3.50	3.68
WCST perseverative error	0.48	0.35	0.01	1.36

*WCST *Wisconsin Card Sorting Task

### Flanker and WCST metrics as predictors of creative thinking

As in Experiment 1, random intercepts and slopes for each participant were extracted to capture individual attentional profiles from the flanker task. Three subject-level metrics were derived: (i) SustainedRT, reflecting baseline RT on congruent trials; (ii) InhibitionCost, which is the RT change for incongruent minus congruent trials; and (iii) FlexibilityCost, which is the RT change for reversed minus congruent trials. A more positive score on the latter two metrics represents a larger cost (i.e., poorer performance on incongruent or reversed trials, respectively, relative to congruent trials). These metrics were used as predictors in the subsequent regression model below.

Whereas Experiment 1 examined RT and accuracy effects separately, Experiment 2 integrated these indices within a single analytic framework to test their combined predictive influence on creative performance, while also including the WCST (perseverative and log-transformed RTs) as a broader measure of cognitive flexibility.

We used four multiple linear regression models to assess whether flanker and WCST metrics predicted CRAT performance (Model 1), AUT fluency (Model 2), AUT originality (Model 3) and AUT flexibility (Model 4). All model results are presented in Table 15. Model 1 was significant, *F*(9, 39) = 17.20, *p* < .001, with 75.2% of the variance explained (adjusted *R*² = 0.752). Significant predictors included: (i) InhibitionCost (*b* = − 0.03, *p* = .026); (ii) SustainedRT (*b* = − 0.06, *p* = .010); (iii) Accuracy Incongruent (*b* = 0.06, *p* = .008); (iv) Accuracy Reversed (*b* = 0.07, *p* = .009); and (v) WCST perseverative error (log) (*b* = − 0.08, *p* < .001). These findings suggest that better attentional inhibition (lower costs on incongruent trials) and enhanced cognitive flexibility across both the flanker task (lower costs on reversed trials) and WCST (faster RTs) strongly predict CRAT accuracy. Model 2 was not significant, *F*(9, 39) = 0.94, *p* = .50, and did not explain meaningful variance (adjusted *R*² = –0.04). None of the predictors reached significance (all *p*s > 0.30), indicating that attentional inhibition and cognitive flexibility measures did not predict AUT fluency. Model 3 was significant, *F*(9, 39) = 10.65, *p* < .001, explaining 64.4% of the variance (adjusted *R*² = 0.644), suggesting that better attentional inhibition (lower attentional costs, especially under reversed conditions) and enhanced cognitive flexibility (faster RTs in the WCST) predicted higher AUT originality scores. Model 4 was significant, *F*(8, 40) = 2.30, *p* = .039, with 31.5% of the variance explained (Adjusted *R*² = 0.178). Although no individual predictors reached conventional significance, faster responses on reversed trials (indexed by a lower FlexibilityCost; *p* = .08) tended to predict increased AUT flexibility.

**Table 15:** Linear regression models predicting CRAT accuracy, AUT fluency, AUT originality and AUT flexibility outcomes from accuracy and RT metrics derived from both the flanker and WCST

Model	Predictor	B	SE	t	*p*
Model 1 (CRATs)	(Intercept)	0.65	0.07	9.12	< 0.001***
	SustainedRT	–0.01	0.02	–0.75	0.458
	InhibitionCost	–0.03	0.01	–2.31	0.026*
	FlexibilityCost	–0.01	0.02	–0.63	0.532
	Baseline accuracy	0.06	0.02	2.71	0.010*
	Accuracy incongruent	0.06	0.02	2.79	0.008**
	Accuracy reversed	0.07	0.03	2.73	0.009**
	WCST perseverative error	–0.13	0.09	–1.52	0.138
	WCST RT (log)	–0.08	0.02	–4.71	< 0.001***
Model 2 (AUT fluency)	(Intercept)	6.09	1.54	3.96	< 0.001***
	SustainedRT	–0.04	0.42	–0.10	0.920
	InhibitionCost	0.16	0.31	0.52	0.604
	FlexibilityCost	0.06	0.44	0.14	0.887
	Baseline accuracy	–0.27	0.45	–0.61	0.544
	Accuracy incongruent	–0.01	0.48	–0.02	0.983
	Accuracy reversed	–0.20	0.52	–0.38	0.706
	WCST perseverative error	–2.20	1.74	–1.27	0.214
	WCST RT (log)	–0.27	0.34	–0.80	0.426
Model 3 (AUT originality)	(Intercept)	5.65	1.21	4.67	< 0.001***
	SustainedRT	–0.27	0.33	–0.83	0.414
	InhibitionCost	–0.40	0.24	–1.69	0.099
	FlexibilityCost	–0.23	0.35	–0.65	0.518
	Baseline accuracy	1.16	0.36	3.25	0.002**
	Accuracy incongruent	0.76	0.39	1.94	0.060
	Accuracy reversed	1.13	0.43	2.65	0.012**
	WCST perseverative error	–0.98	1.45	–0.67	0.505
	WCST RT (log)	–0.66	0.28	–2.36	0.023*
Model 4: AUT flexibility	Intercept	5.639	1.340	4.21	< 0.001***
	SustainedRT	–0.001	0.003	–0.22	0.827
	InhibitionCost	–0.027	0.023	–1.15	0.256
	FlexibilityCost	–0.020	0.011	–1.77	0.084
	Baseline accuracy	0.192	0.259	0.74	0.462
	Accuracy incongruent	0.620	0.502	1.24	0.224
	Accuracy reversed	0.774	0.479	1.62	0.114
	WCST perseverative error	–1.380	1.194	–1.16	0.255
	WCST RT (log)	0.003	0.006	–0.99	0.329

### Mediation models

Descriptive analysis of the problem-solving strategy scores revealed that of the total correct responses to the CRAT, 47.8% were reported as being solved via a more insight-based approach, while 52.2% were reported as being solved using a more analytic-based approach. Prior to conducting our mediation analyses, we explored the influence of our 30 s time limit on the strategies that participants reported using to solve CRAT items, by conducting a Spearman’s correlation on trial-level data. This correlation revealed a significant negative association between CRAT reaction times and strategy ratings (*r*_s_ (782) = − 0.41, *p* < .001), such that faster responses were significantly more likely to be categorised as being solved via insight, whereas longer responses were more likely to be categorised as being solved via analysis.

**Fig. 3:**
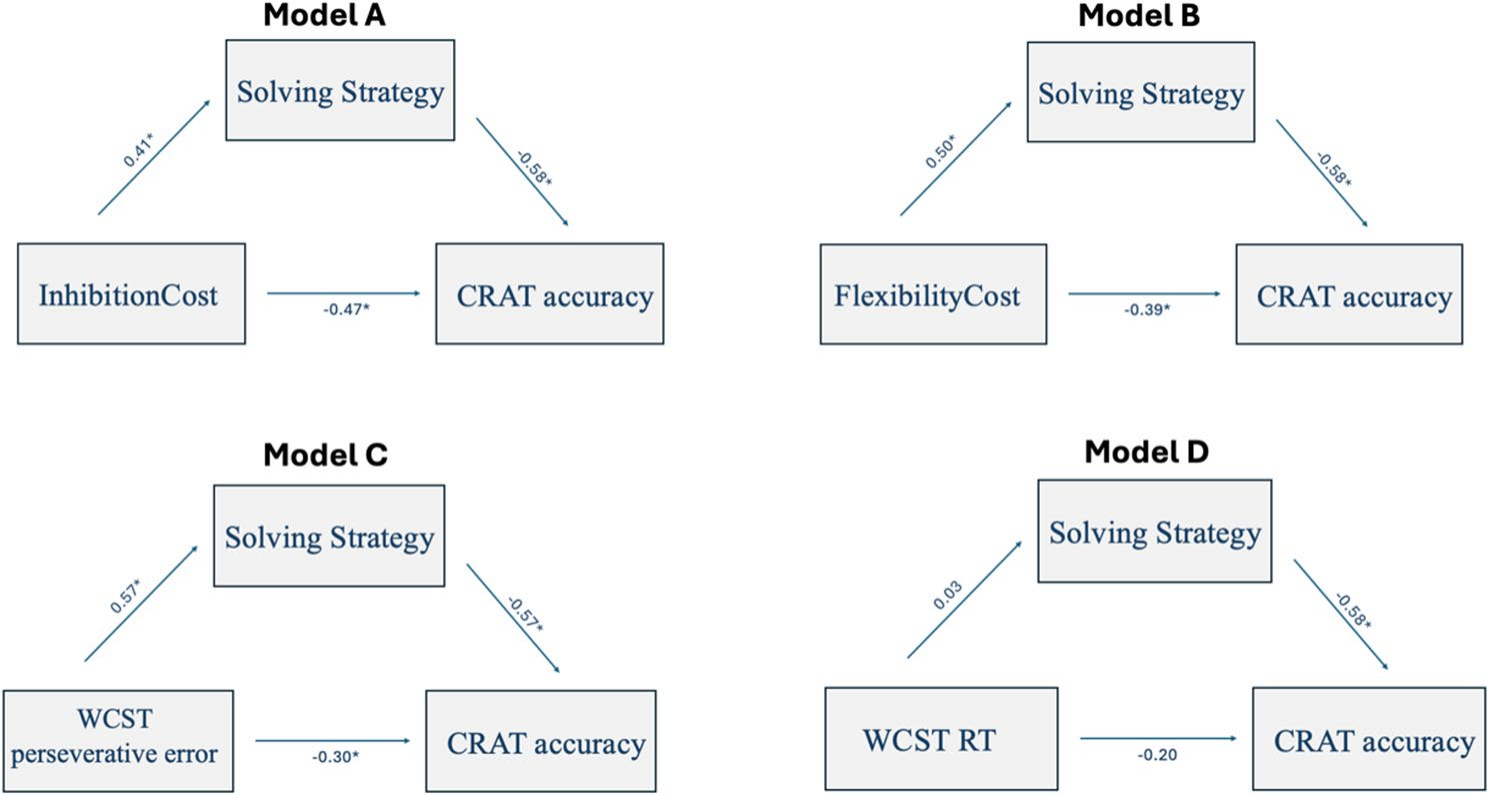
Mediation diagrams illustrating the indirect and direct effects of attentional inhibition and cognitive flexibility on CRAT accuracy via solving strategy. Note. Each panel represents a separate mediation model: (Model A) InhibitionCost, (Model B) FlexibilityCost, (Model C) WCST perseverative error, and (D) WCST RT (log transformed). Values represent standardized path coefficients (*β*). Asterisks (*) denote statistically significant paths (*p* < .05). All models used nonparametric bootstrapping with 5,000 iterations and 95% percentile-based confidence intervals.

We next conducted four separate mediation analyses to examine whether problem solving strategy mediated the relationship between attentional inhibition and convergent thinking as well as between cognitive flexibility and convergent thinking, in both cases with convergent thinking being measured in terms of CRAT accuracy (see Fig. 3). In these analyses, a lower problem-solving strategy score reflects a more analytic-based approach to solving CRAT items, whereas a higher problem-solving strategy score reflects a more insight-based approach.

We first tested whether CRAT solving strategy mediated the relationship between flanker InhibitionCost (reflecting attentional inhibition) and CRAT accuracy. The average causal mediation effect (ACME) was significant, indicating partial mediation, − 0.024, 95% CI [− 0.042, − 0.010], *p* = .002. The average direct effect (ADE) remained significant, ADE = − 0.047, 95% CI [− 0.067, − 0.030], *p* < .001, suggesting that flanker InhibitionCost influenced CRAT accuracy both directly and indirectly via strategy use. The total effect was significant, − 0.071, 95% CI [− 0.090, − 0.060], *p* < .001. Approximately 34.1% of the total effect was mediated through strategy. These results indicate that better attentional inhibition (lower interference costs) was associated with higher CRAT accuracy, and that this relationship was partially explained by participants’ problem-solving strategy (more analytic).

A similar pattern emerged for FlexibilityCost. The mediation analysis revealed a significant indirect effect of FlexibilityCost (reflecting cognitive flexibility) on CRAT accuracy through solving strategy, − 0.044, 95% CI [− 0.081, − 0.020], *p* < .001. The direct effect also remained significant − 0.060, 95% CI [− 0.092, − 0.030], *p* < .001, and the total effect was robust, − 0.104, 95% CI [− 0.136, − 0.081], *p* < .001. The proportion of the total effect mediated was 42.3%. These results suggest that enhanced cognitive flexibility (reflected by lower RT costs) predicts creative problem solving (higher CRAT accuracy) by encouraging a more analytic strategy when solving CRAT items.

Next, we examined whether solving strategy mediated the relationship between WCST perseverative error (higher score = higher perseverative errors) and CRAT accuracy. WCST perseverative error was negatively associated with both solving strategy and CRAT performance. The indirect effect was significant, − 0.215, 95% CI [–0.088, − 0.390], *p* < .001. The direct effect approached significance, − 0.196, 95% CI [–0.012, − 0.360], *p* = .060, while the total effect was significant, = − 0.411, 95% CI [–0.300, − 0.540], *p* < .001. The proportion of the total effect mediated through solving strategy was 52.3%. These results suggest that enhanced cognitive flexibility (as indexed by lower WCST perseverative error scores) predicted higher CRAT accuracy in part because those individuals were more likely to report using analytical solving strategies.

Finally, we tested whether solving strategy mediated the relationship between WCST response time and CRAT accuracy. The mediation pathway was not significant, − 0.007, 95% CI [− 0.032, 0.020], *p* = .530. However, the direct effect remained significant, − 0.086, 95% CI [− 0.113, − 0.060], *p* < .001, and the total effect was significant, − 0.093, 95% CI [− 0.111, − 0.080], *p* < .001. Only 7.8% of the total effect was mediated by solving strategy. These findings indicate that slower WCST performance (indicative of lower cognitive flexibility) predicted lower CRAT accuracy (indicative of lower convergent thinking) independently of strategy use.

## Discussion

Consistent with Hypothesis 1, faster RTs on incongruent flanker trials (lower interference costs) and higher accuracy on incongruent and reversed conditions (indicating greater attentional inhibition and cognitive flexibility, respectively), predicted higher CRAT accuracy. These findings replicate and extend on Experiment 1, highlighting the joint positive influence of attentional inhibition and cognitive flexibility on convergent thinking.

Partially supporting Hypothesis 2, less attentional interference in the reversed flanker condition (lower interference costs) positively predicted AUT originality, but not AUT fluency or AUT flexibility. This finding suggests that attentional inhibition more strongly influences the originality component of divergent thinking rather than the sheer quantity or flexibility of generated ideas.

Hypothesis 3 was partially supported. Although higher cognitive flexibility (indexed by lower WCST perseverative error) showed a positive trend toward predicting CRAT accuracy, this was not statistically significant. However, better cognitive flexibility (indexed by lower WCST RT) emerged as a strong, significant predictor of CRAT accuracy. This supports the notion that cognitive flexibility, especially the efficiency of adapting to changing rules, contributes meaningfully to convergent thinking. 

Partially supporting Hypothesis 4, we found a trend for WCST perseverative error to predict AUT originality scores negatively, albeit non-significantly, while no significant prediction was found for AUT fluency or AUT flexibility. These results suggest that broader cognitive flexibility may partially support the originality aspect of divergent thinking but has limited influence on idea fluency or flexibility.

Exploratory analyses examining problem-solving strategy in the CRAT revealed that strategy use did partially mediate the relationships between attentional inhibition and CRAT accuracy, as well as between cognitive flexibility and CRAT accuracy. Mediation models indicated that lower inhibition and flexibility costs (Models A and B) and lower WCST perseverative error (Model C) predicted higher CRAT accuracy in part by encouraging more analytic problem-solving strategies. Notably, strategy use mediated approximately 34–52% of these effects, suggesting that individual differences in attentional inhibition and cognitive flexibility influence creative outcomes partly through their impact on problem-solving approaches (i.e., supporting increased analytic processing). Conversely, the mediation effect of solving strategy on the relationship between WCST RT and CRAT accuracy was not significant, indicating that cognitive flexibility, as indexed by RT, predicts CRAT performance independently of strategy. Finally, our exploratory correlational analyses support the notion that insight solutions tend to occur more rapidly than analytic solutions in the CRAT.

## General discussion

Across two experiments, we investigated how attentional inhibition and cognitive flexibility relate to creative cognition, with a particular focus on convergent and divergent creative thinking. Drawing on prior research linking executive processes to creative cognition [10, 16, 19], we tested whether individuals with superior capacities to inhibit distractions and to adapt flexibly to rule changes would perform better on creative tasks that require either convergent thinking or divergent thinking. Additionally, we examined whether the relationship between attentional inhibition and cognitive flexibility for creative outputs on a convergent thinking task is mediated by an individual’s problem-solving strategy in terms of whether this is more insight-based versus more analytic in nature [67, 68].

Experiment 1 provided initial support for some of our key hypotheses by demonstrating that higher attentional inhibition and cognitive flexibility (with these two attentional facets being measured using a modified flanker task), predicted higher performance on both convergent (the CRAT) and divergent (the AUT) thinking tasks. Specifically, higher cognitive control under incongruent conditions (requiring attentional inhibition) and reversed conditions (requiring both attentional inhibition and cognitive flexibility), was associated with increased creative performance across both convergent and divergent thinking tasks. For divergent thinking, these effects were most pronounced for AUT originality, suggesting that the *quality* of ideation is particularly influenced by attentional inhibition and cognitive flexibility, rather than the quantity or flexibility of ideas.

Experiment 2 replicated and extended these findings by incorporating within the study a well-established measure of broad cognitive flexibility (the WCST) and a subjective assessment of problem-solving strategy for the convergent thinking task (the CRAT). Individuals who exhibited higher attentional inhibition and cognitive flexibility on the flanker task as well as higher cognitive flexibility on the WCST were more likely to solve CRAT items correctly and were also more likely to report using analytic-based strategies. WCST performance also accounted for unique variance in CRAT scores beyond flanker-based measures, reinforcing the importance of cognitive flexibility in convergent thinking. In relation to the AUT as a measure of divergent thinking, although greater cognitive flexibility on the flanker task positively predicted AUT originality, neither attentional inhibition nor cognitive flexibility significantly predicted AUT fluency (i.e., the number of ideas generated) or AUT flexibility (i.e., the range of categories). These latter findings are consistent with those of Experiment 1 as well as with prior research showing that performance indices relating to the AUT (e.g., fluency, originality and flexibility) may depend on dissociable cognitive processes [9, 26].

### Theoretical contribution

Our findings help to clarify the distinct roles of attentional inhibition and cognitive flexibility in convergent thinking and divergent thinking, respectively. First, the predictive power of inhibition costs on CRAT performance across both experiments reinforces the view that convergent thinking relies heavily on attentional inhibition, including the capacity to suppress dominant or misleading associations and to reconfigure unhelpful problem representations. The potency of enhanced attentional inhibition in predicting CRAT accuracy aligns with models proposing that attentional inhibition is essential for overriding habitual or automatic responses in favour of novel yet task-relevant alternatives [12, 23, 45]. Such findings lend weight to the “business-as-usual” view of creativity, which suggests that creative thinking, particularly in convergent thinking tasks, engages the same top-down executive processes recruited for goal-directed problem solving more broadly [33].

Second, our measures of cognitive flexibility (indexed by performance on reversed flanker trials as well as by performance on the WCST) highlight the critical importance of abilities at mental set shifting for both convergent thinking and, to a lesser extent, divergent thinking. In our convergent thinking task, cognitive flexibility was associated with better CRAT performance and increased use of analytic strategies, supporting theoretical accounts that emphasise controlled, analytical search processes as a key mechanism underlying convergent thinking [69]. These observations also align with dual-process models positing that convergent problem solving is often guided by deliberate, rule-based processes, in contrast to the more associative mechanisms typically invoked in divergent thinking [24]. The ability to abandon ineffective solution ideas and to switch to alternative solution options seems to be central to problem solving in convergent thinking tasks, and our results suggest that cognitive flexibility may support such adaptive shifts.

Third, our mediation analyses provide a deeper understanding of how attentional inhibition and cognitive flexibility shape convergent thinking. Rather than exerting uniformly direct effects, both of these control processes (attentional inhibition and cognitive flexibility) predicted strategic engagement, specifically, a greater likelihood of using analytic rather than insight-based problem-solving strategies. This finding aligns with the “business-as-usual” view of creativity, which posits that creative problem solving in convergent thinking tasks relies on the same top-down executive processes involved in general goal-directed cognition [33]. From this perspective, individuals with higher executive control may be more capable of engaging and sustaining systematic, rule-based search strategies, allowing them to navigate the problem space efficiently without prematurely abandoning effortful reasoning. The use of the reversed flanker condition, designed to tax inhibition and rule switching simultaneously, was seen to be especially predictive of performance in our convergent thinking task. The sensitivity to moment-to-moment demands on attentional control, captured by our FlexibilityCost metric, seemed to complement the broader, ecologically valid WCST measure. That both attentional inhibition and cognitive flexibility predicted analytic strategy use, and that increased analytic strategy use partially mediated the effects of these attentional variables on CRAT accuracy, supports the view that creative cognition depends not only on attentional control capacities themselves, but also on how attentional processes like inhibition and flexibility are strategically deployed to align with the type of creativity task at hand.

Finally, although the effects of attentional inhibition and cognitive flexibility on divergent thinking (i.e., AUT performance) were more modest than for the CRAT, exploratory analyses revealed that better attentional inhibition (lower InhibitionCost in Experiment 1 only) and cognitive flexibility (faster RTs on the WCST) were associated with higher AUT originality scores, but not AUT fluency or AUT flexibility scores. This pattern of results suggests that attentional inhibition and cognitive flexibility (WCST only) may be more critical for the evaluation of original ideas in the AUT, rather than for the generation of ideas or for the flexibility of ideas. These findings are consistent with previous research showing that generating *original* ideas requires the ability to suppress dominant or obvious associations during stages of idea evaluation, as well as the capacity to shift flexibly between modes of idea generation and idea evaluation [16, 19].

In summary, our findings converge on the view that creative cognition, particularly in convergent tasks like the CRAT, relies on top-down control processes. Our observation that attentional inhibition and cognitive flexibility predict CRAT accuracy by promoting the use of analytic problem-solving strategies aligns closely with the “business-as-usual” account of creativity, which posits that convergent thinking draws upon the same controlled, analytic and deliberate processes that support non-creative problem solving [33]. In this sense, our results indicate that successful convergent thinking depends on the effective engagement of executive functions (i.e., attentional inhibition and cognitive flexibility) that enable focused, goal-directed idea generation and evaluation.

In contrast, our results from a divergent thinking task (the AUT) reveal a more selective pattern: only attentional inhibition predicted the originality of ideas, potentially by allowing for the inhibition of distracting or irrelevant information when evaluating ideas, whereas neither attentional inhibition nor cognitive flexibility predicted other AUT outcomes more centred around idea generation (AUT fluency and AUT flexibility). This pattern suggests that although the evaluative aspect of divergent thinking (AUT originality) benefits from attentional control, the generative dimensions (AUT fluency, AUT flexibility) are potentially governed more by Type 1 processes that are associative and spontaneous in nature, which are thought to facilitate the broad semantic activation that supports idea generation [24].

Taken together, our findings in relation to convergent thinking align most consistently with a “business-as-usual” account of creative cognition [33]. In contrast, our findings for divergent thinking invite a more integrated or dual-process interpretation [24, 29] to explain the selective patterns whereby ideation may depend upon additional associative processes to support generative dimensions. Indeed, broader frameworks of creativity, such as the dual-process model [24], describe how analytic and associative processes may interact during creative thinking. However, since we did not directly measure Type 1 associative processes in our experiments, our data primarily speak to the controlled, analytic aspects of the dual-process model.

### Limitations and future directions

Several factors may limit the interpretation and generalisability of the current findings. First, our assessment of problem-solving strategy relied on retrospective self-reports following each CRAT item. Although this approach offers ecological validity and participant-level reports of problem-solving experiences, it is vulnerable to some limitations. For example, participants may lack access to the cognitive processes that underlie their solutions, particularly for insight-driven responses that emerge spontaneously, thus future studies may incorporate other techniques such as think-aloud protocols, eye-tracking, or mouse-tracking paradigms to capture a more dynamic and objective report of strategy use in real time. These methods may allow for finer-grained temporal analysis of when and how shifts between analytic and associative strategies occur during problem solving.

A further methodological consideration is the 30 s time limit that we employed for each CRAT item. This window is longer than the 15–20 s used in prior work [57] and may have altered the balance of cognitive processes that were engaged. Whereas short deadlines tend to capture rapid, insight-based solutions that arise spontaneously, longer deadlines tend to capture more analytic-based solutions, given that participants have more time to persist with deliberate, analytic strategies, such as hypothesis testing, systematic search, and sustained monitoring [70]. The foundational paper that introduced normative data for CRAT items [22] found that when participants solved the item correctly with a 30 s time limit, 56% of items were approached via insight and 44% of items were solved analytically. In support, Sandkühler and Bhattacharya [71] identified a 30 s time limit as the “sweet spot” for capturing a robust balance of both insight and analytic solving strategies, whereby windows that are too short (i.e., less than 10 s) or windows that are too long (i.e., 60 s) will primarily capture either insight or analytic strategies, respectively.

Our data support this latter interpretation of a strategic shift toward analysis over time. Among correctly solved items, 52.2% were reported as being solved via analysis, and 47.8% were reported as being solved via insight. Furthermore, a trial-level Spearman correlation revealed a robust negative association between response time (i.e., the time taken to submit a solution) and strategy ratings. This finding confirms that as participants moved closer to the 30 s limit, they were significantly more likely to employ an analytic approach rather than an insight-based one. Whilst the longer timeframe that was available to participants likely improved overall solution rates, we argue that this time limit did not necessarily encourage a specific solving strategy but instead provided participants with sufficient time to solve via insight or via analysis (as evidenced by the balanced strategy approaches reported above).

Importantly, although our design focused on attentional mechanisms, creative thinking is a multifaceted construct shaped by affective, motivational, and dispositional variables. For example, openness to experience, tolerance of ambiguity, and intrinsic motivation are all known to influence creative engagement but were not assessed in the present study [72, 73]. Neurocognitive models such as the default–executive coupling hypothesis [15] and the dual pathway to creativity model [35] emphasise the role of affect and spontaneous thought in modulating cognitive control during creativity. Future studies would benefit from integrating trait-level assessments, state-based manipulations (e.g., mood induction, reward sensitivity), and neuroimaging methods to examine how executive processes interact with affective and motivational systems in real time. A further limitation concerns the demographic characteristics of our sample. Participants across both experiments were predominantly young university students (mean age = 24.2 years in Experiment 1; mean age = 27.5 years in Experiment 2), White British (74.6% in Experiment 1; 81.6% in Experiment 2), and female (74.5% in Experiment 1; 71.4% in Experiment 2). This reliance on a young and predominantly female university student sample may constrain the generalisability of our findings.

Finally, although our task battery included measures of both convergent thinking (using the CRAT) and divergent thinking (using the AUT), the latter was limited to assessments of idea fluency (the number of ideas generated), idea originality (the uniqueness or novelty of those ideas) and idea flexibility (the range of idea categories). This AUT scoring procedure, although widely used, raises questions about what aspects of creativity are truly captured. Fluency, in particular, has been criticised as a potentially weak proxy for creativity, as generating many ideas does not necessarily equate to creative quality or usefulness. Indeed, our findings indicated that executive control measures were more closely related to AUT originality than AUT fluency or AUT flexibility, suggesting that the quality rather than the quantity of ideas or idea categories is more reflective of (divergent) creative cognition under executive demands. Future research should consider incorporating additional scoring dimensions such as elaboration (the detail or development of ideas), usefulness, or appropriateness to provide a richer, multidimensional assessment of divergent thinking.

## Conclusion

Our findings help to clarify the distinct roles of attentional inhibition and cognitive flexibility in creative cognition. Across two experiments, we demonstrate that attentional inhibition and cognitive flexibility predict convergent thinking, as measured by CRAT accuracy, both directly and via their influence on strategic problem-solving approaches. Individuals with greater inhibitory control and set-shifting capacity were more likely to engage in analytic-based strategies, which in turn supported successful problem solving in a convergent thinking task. These results provide empirical support for the business-as-usual view, by highlighting the role of goal-directed control in convergent thinking. Although the influence of attentional inhibition and cognitive flexibility on divergent thinking was seen to be more modest, the effect of these attentional factors on the originality measure of divergent thinking seems to support the view that attentional control is more relevant for the evaluation of ideas, rather than for idea generation. Overall, our findings suggest that whilst attentional inhibition and cognitive flexibility demonstrate a stronger and more consistent predictive relationship with convergent thinking performance, aligning with the business-as-usual model, divergent thinking performance invites a more integrated interpretation.

## Data Availability

All datasets and analysis scripts have been made publicly available at The Open Science Framework ( [https://osf.io/ve2my/overview?view_only=634da9164b9249929666b5779f6fa05d](https:/osf.io/ve2my/overview?view_only=634da9164b9249929666b5779f6fa05d) ). This project was not preregistered.
